# Targeting EP2 Receptor Improves Muscle and Bone Health in Dystrophin^−/−^/Utrophin^−/−^ Double-Knockout Mice

**DOI:** 10.3390/cells14020116

**Published:** 2025-01-14

**Authors:** Xueqin Gao, Yan Cui, Greg Zhang, Joseph J. Ruzbarsky, Bing Wang, Jonathan E. Layne, Xiang Xiao, Johnny Huard

**Affiliations:** 1 Linda and Mitch Hart Center for Regenerative and Personalized Medicine, Steadman Philippon Research Institute, Vail, CO 81657, USA; jruzbarsky@thesteadmanclinic.com (J.J.R.); jonathan.layne@cuanschutz.edu (J.E.L.); 2 Department of Orthopaedic Surgery, McGovern Medical School, University of Texas Health Science Center at Houston, Houston, TX 77030, USA; yacui2@mdanderson.org (Y.C.); greg00010@gmail.com (G.Z.); 3 Vascular Medicine Institute, Division of Cardiology, Department of Medicine, University of Pittsburgh, Pittsburgh, PA 15260, USA; bingwang@pitt.edu; 4 Pittsburgh VA Healthcare System, Pittsburgh, PA 15240, USA; 5 Glassell School of Art, The Museum of Fine Arts, Houston, TX 77006, USA; xiangxiao6363@gmail.com

**Keywords:** muscular dystrophy, EP2, EP4, PGE2, bone microarchitecture, heterotopic ossification, PF04418948

## Abstract

Duchenne muscular dystrophy (DMD) is a severe genetic muscle disease occurring due to mutations of the dystrophin gene. There is no cure for DMD. Using a dystrophin^−/−^utrophin^−/−^ (DKO-Hom) mouse model, we investigated the PGE2/EP2 pathway in the pathogenesis of dystrophic muscle and its potential as a therapeutic target. We found that Ep2, Ep4, Cox-2, 15-Pgdh mRNA, and PGE2 were significantly increased in DKO-Hom mice compared to wild-type (WT) mice. The EP2 and EP4 receptors were mainly expressed in CD68^+^ macrophages and were significantly increased in the muscle tissues of both dystrophin^−/−^ (mdx) and DKO-Hom mice compared to WT mice. Osteogenic and osteoclastogenic gene expression in skeletal muscle also increased in DKO-Hom mice, which correlates with severe muscle heterotopic ossification (HO). Treatment of DKO-Hom mice with the EP2 antagonist PF04418948 for 2 weeks increased body weight and reduced HO and muscle pathology by decreasing both total macrophages (CD68^+^) and senescent macrophages (CD68^+^P21^+^), while increasing endothelial cells (CD31^+^). PF04418948 also increased bone volume/total volume (BV/TV), the trabecular thickness (Tb.Th) of the tibia trabecular bone, and the cortical bone thickness of both the femur and tibia without affecting spine trabecular bone microarchitecture. In summary, our results indicate that targeting EP2 improves muscle pathology and improves bone mass in DKO mice.

## 1. Introduction

Duchenne muscular dystrophy (DMD) is a severe genetic muscle disease occurring due to mutations of the dystrophin gene and loss of dystrophin expression. It affects 1 in 3000 boys [1,2]. The manifestations of DMD are severe, and patients die in their third or fourth decades. Currently, there is no cure. Dystrophin^−/−^/utrophin^−/−^ (DKO-Hom) is a mouse model that recapitulates the disease’s clinical manifestation more closely than dystrophin^−/−^ mice (Mdx), with more severe muscle histopathology including muscle necrosis, fibrosis, fat infiltration, heterotopic ossification (HO), kyphosis, and a short lifespan [3,4,5,6,7]. Previously, we have shown that DKO-Hom mice also exhibit a spectrum of musculoskeletal abnormalities, including bone osteopenia [8]. Bone osteopenia was determined to be a secondary consequence of the muscle disease, with mice demonstrating declines in osteoblasts, osteoclasts, and osteocytes [9]. Furthermore, we also found impaired fracture healing in DKO-Hom mice [10]. DMD patients also have increased fracture risk, especially vertebrate fractures, irrespective of prior steroid treatment [11,12].

Restoring functional dystrophin is the fundamental solution for facilitating a cure. Previous studies using different AAV vectors to deliver micro, mini, or full-length dystrophin genes have shown that they express functional dystrophin and improve muscle pathology, enhance growth and mobility, inhibit spine deformation, and extend the lifespan of mice [13,14,15,16,17,18,19]. More recently, ELEVIDYS, an AAV-based gene therapy (micro-dystrophin) has been approved by the FDA for the treatment of ambulatory pediatric patients, aged 4 through 5 years, with DMD with a confirmed mutation in the DMD gene [20]. CRISPR/Cas9 gene-editing technology renders new strategies to restore functional endogenous dystrophin by guided RNA-mediated excision of mutant dystrophin genes including exon 23. It has been successfully demonstrated to restore functional dystrophin protein in cardiac muscle and skeletal muscle and to improve muscle strength using different mouse models [21,22]. Using a non-homologous end-joining (NHEJ) CRISPR/cas 9 editing technology to remove mutant exon 23 in DKO-Hom mice was also reported. Although this approach can restore dystrophin protein expression, it only induced dystrophin expression at 5.7% of the level of WT mice. In addition, some unwanted cutting sites were identified in the genome and caused safety concerns [23]. More recently, base and prime editing was also used to edit the mutant dystrophin gene to restore its function and demonstrated high efficacy [24,25]. Cell therapy has also been explored for the treatment of DMD. The systemic transplantation of different stem cells through tail vein injection to DKO-Hom mice has been shown to improve motor function and muscle pathology and increase the lifespan of the treated mice [26,27,28,29].

Targeting abnormally expressed genes in DMD has also been shown to improve muscle histopathology. Sarcolipin (SLN) is an inhibitor of the sarco/endoplasmic reticulum (SR) Ca^2+^ ATPase (SERCA) and is abnormally elevated in the muscle of DMD patients and animal models. Either knocking down the SLN, using genetic approaches, or AAV-SLN siRNA attenuates muscle pathology and improves diaphragm, skeletal muscle, and cardiac function [30]. IL6 was also found to be significantly increased in DKO-Hom mice; IP injection of the IL6 antibody MRL6-1 significantly increased embryonic myosin heavy chain and muscle diameter and reduced fibrosis via decreasing the phosphorylated signal transducer and activator of transcription 3 (pSTAT3) [31].

Despite all the above research advancements, the current treatment of DMD patients is still limited, and no cure is available. Inflammation remains a profound pathological change in the muscle of muscular dystrophy [4,5,9]. Hence, anti-inflammation is equally important in DMD treatment, in addition to the restoration of the dystrophin gene. Steroids targeting inflammation are the only palliative therapy available but have side effects, including increasing fracture risk after long-term treatment [32,33,34]. Therefore, identifying signaling pathways that contribute to muscle pathology is necessary.

The cyclooxygenase 2 (COX2)/prostaglandins E2 (PGE2) signaling pathway is one of earliest recognized inflammatory signaling pathways during inflammation [35,36]. COX-2 is a rate-limiting enzyme that catabolizes arachidonic acid to generate prostaglandin H (PGH) and is subsequently converted by microsomal prostaglandin E synthase 1 and 2 (mPGES 1,2) into PGE2 and other prostaglandin signal molecules. PGE2 binds to the prostaglandin E receptors 1-4 (EP1-4) via different G proteins, produces different effects in different organs, and is responsible for a variety of physiological functions [37,38,39,40,41,42,43,44]. PGE2 binds to EP 2 and 4 via G proteins, activates adenosine cyclase (AC), increases CAMP, and subsequently activates protein kinase A (PKA). When PGE2 binds to EP3, it activates Gi protein and inhibits CAMP production. When PGE2 binds to EP1, it activates Gq and subsequently activates phospholipase C and increases cytosol Ca^2+^ [45].

COX2 plays an important role in muscle regeneration in a time-dependent manner [46]. COX2 activity is also critical for muscle hypertrophy after overloading. The COX2 selective inhibitor, NS398, decreased macrophage infiltration and proliferation [47]. PGE2 was also demonstrated to be essential for muscle stem cell function and expansion, while promoting muscle regeneration via binding to EP4 receptors. This mechanism involves PGE2 binding to the EP4 receptor and activating cAMP/CREB via the Nurr 1 transcription factor [48]. Inhibition of the Notch signaling pathway via N-[N-(3,5-difluorophenacetyl-L-alanyl)]-S-phenylglycine tertial butyl ester (DAPT) promotes the fusion of human muscle progenitors in vitro and improves their engraftment in the tibialis anterior muscle of immune-deficient mice. Gene expression analysis revealed that DAPT severely down-regulates EP2 in human muscle progenitors in differentiation condition. Functional analysis suggests that Notch signaling inhibits differentiation and promotes self-renewal of human muscle progenitors via PGE2/EP2 signaling in a cAMP/PKA-independent manner [49]. A very early study demonstrated that muscle strips from a human DMD patient release PGE2 more than control muscles [50], but no study has investigated the role of COX2/PGE2 in the pathogenesis of Duchenne muscular dystrophy.

Given that little information is known regarding the role of the COX2/PGE2/EP2 signaling pathway in the pathogenesis of DMD, the aim of this study was to investigate the role of the prostaglandin E 2/prostaglandin E 2 receptor 2 (PGE2/EP2) signaling pathway, including enzymes that catabolize the production of PGE2 and degrade PGE2 and their receptor’s expression, and its involvement in the development of muscle pathology (including HO) in DKO-Hom mice and to determine if targeting the PGE2/EP2 signaling pathway can improve the pathology of muscular dystrophic muscle and bone health.

## 2. Materials and Methods

### 2.1. Mice Breeding

Dystrophin^−/−^Utrophin^+/−^ (DKO-Het) mice were provided by our collaborator [16] and bred to generate Dystrophin^−/−^ (Mdx) and Dystrophin^−/−^Utrophin^−/−^ (DKO-Hom) mice for the experiments. Genotyping was performed at the time of weaning at 3 weeks after birth. All the experiments were performed according to the Institutional Animal Care and Use Committee by the University of Texas Health Science Center at Houston (Protocol # AWC-15-0073) and Colorado State University (#1234).

### 2.2. PGE2-EP2/4 Signaling and Inflammatory Gene Expression Detection Using 4-Week-Old Mice

Four-week-old C57BL/10J (WT) mice were purchased from Jackson Laboratories. Mdx and DKO-Hom mice were generated by breeding DKO-Het mice and were sacrificed at 4 weeks old to harvest muscle tissues. Gastrocnemius muscles were dissected and frozen in 2-methylbutane in liquid nitrogen on the cork using Cryogel for cryosectioning. Thigh muscle tissues were dissected and immediately stored at −80 °C for later RNA extraction, CDNA synthesis, and quantitative polymerase chain reaction (Q-PCR).

### 2.3. Mice Treatment with EP2 Antagonist PF04418948

Four-week-old DKO-Hom (male and female) mice were treated with the vehicle (*n* = 6) (0.5% *w*/*v* methylcellulose + 0.1% *v*/*v* Tween 80 in purified water) according to the previous literature [51] or 10 mg/kg/day PF04418948 in the vehicle (0.5% *w*/*v* methylcellulose + 0.1% *v*/*v* Tween 80 in purified water) (*n* = 8) (Cat. No. 4818, Tocris, R&D system, Minneapolis, MN, USA) for 2 weeks by oral gavage daily. Mice were then sacrificed. The left-side gastrocnemius muscle tissues were dissected and frozen in 2-methylbutane in liquid nitrogen on the cork for cryosection and staining. Left-side thigh muscles were also dissected and immediately frozen at −80 °C for RNA isolation. The right tibia and femur bone tissues, together with attached intact muscle tissues, as well as the skull and lumbar spine were then dissected and fixed with neutral-buffered formalin (NBF) for 72 h for subsequent micro-CT scanning and histology analysis.

### 2.4. RNA Extraction, cDNA Synthesis, and Q-PCR Analysis

Total RNA was then extracted from the dissected thigh muscle using TRIzol Reagents (Themo-Fisher Invitrogen, Waltham, MA, USA), following the manufacturer’s protocol. Total RNA was dissolved in DNAase/RNAase-free water. RNA concentration was measured with a Tecan 200 Infinite plate reader equipped with Nanoplate. RNA was stored at −80 °C. cDNA synthesis was performed using Iscript Reverse Transcription Supermix (1708841, BioRad, Hercules, CA, USA ) using 1 µg total RNA. cDNA was diluted 1:5 using DNAase/RNAase-free water and stored at −20 °C until use. Q-PCR was performed using the SsoAdvanced Universal SYBR^®^ Green Supermix Q-PCR kit (1725271, BioRad, Hercules, CA, USA). The targets analyzed included Cox1, Cox2,15-Pgdh, prostaglandin-E2 (PGE2) receptor 2 (Ep2), prostaglandin-E2 (PGE2) receptor 4 (Ep4), cluster differentiation 68 (CD68), runt-related transcription factor 2 (Runx2), osterix (Osx), cathepsin k (Ctsk), tartrate-resistant acid phosphatase (Trap), follistatin (Fst), myostatin (Mstn), and fibronectin type III domain containing 5 (Fndc5). All primers were designed using Primer 3 Input [52,53,54]. The primers used in this study are listed in Table 1.

### 2.5. Measurement of Prostaglandin 2 (PGE2) Using Enzyme-Linked Immunosorbent Assay (ELISA)

A portion of the thigh muscle tissues from 4-week-old mice of different genotypes were homogenized using a buffer, as suggested by Cayman Chemical, to measure PGE2 using the Cayman ELISA kit (Item No. 514010, Cayman Chemical, Ann Harbor, MI, USA) following the manufacturer’s protocol and normalized to protein concentration. Protein concentration was measured with Pierce™ BCA Protein Assay Kits (catalog number: 23225, Thermo Fisher Scientific, Waltham, MA, USA) following the manufacturer’s protocol.

### 2.6. Immunofluorescence Staining

For 4-week-old mice muscle tissues, we performed CD68/EP2 and CD68/EP4 double staining for gastrocnemius muscle to detect macrophage, EP2, and EP4 expression. For PF04418948 or vehicle-treated mice, we performed CD68/P21, Gr-1/P21, and CD31/P21 double immunofluorescent staining to detect inflammatory cells and endothelial cells after treatment. Briefly, cryosections were cut to 8 µm thickness using Leica CryoStat (Model CM 1950, Leica Biosytems, Nussloch Gmbh, Nussloch, Germany) and stored in a −80 °C freezer. Section slides were taken out from the −80 °C freezer and dried for 10 min and then fixed with 4% paraformaldehyde for 8 min and washed three times with phosphate-buffered saline (PBS) for further immunofluorescence staining, as previously described [55]. Briefly, after blocking with 5% donkey serum (017-000-121, Jackson ImmunoResearch Laboratories Inc., West Grove, PA, USA) in PBS, the slides were incubated with primary antibodies overnight at 4 °C. The dilution of primary antibodies used for this study included rat anti-CD68 (AB53444, 1:100 dilution, Abcam, Cambridge, UK), rabbit anti-EP4 Receptor (C-Term) Polyclonal Antibody (Item No. 101775, 1:50 dilution, Cayman Chemical, Ann Arbor, MI, USA), Recombinant Anti-Prostaglandin E Receptor EP2/PTGER2 antibody [EPR8030(B)] (ab167171, 1:250 dilution, Abcam, Cambridge, UK), rabbit anti-P21 (ab188224, 1:400 dilution, Abcam, Cambridge, UK), rat anti-Gr-1 (BD557445, 1:100, BD), and rat anti-CD31 (BD553370, BD 1:300). After washing with PBS three times for 5 min each, the slides were incubated with secondary antibodies based on the primary antibodies used for each stain for 2 h at ambient temperature. The secondary antibodies used were donkey anti-rat-488 (712-745-153, Jackson ImmunoResearch, Laboratories Inc., West Grove, PA, USA 1:200) and donkey anti-rabbit-594 (711-585-152, 1:200 dilution, Jackson ImmunoResearch Laboratories Inc). After the secondary antibody incubation and another 3 washes, 4′,6-diamidino-2-phenylindole (DAPI) was used to reveal the nuclei. Immunofluorescence images were captured using Nikon-Ni microscope (Nikon Instruments Inc., Melville, NY, USA) using 200× magnification, and positive cell numbers were counted using Image J (Ij154-win-java8).

### 2.7. Muscle Histology

H&E staining was performed on cryosections of gastrocnemius muscle tissues using AnaTech Hematoxylin Extra Strength and Eosin Y according to the manufacturer’s protocol. Von Kossa staining was performed using an IHC World protocol for the detection of HO formation in the muscle tissues (https://www.ihcworld.com/_protocols/special_stains/von_kossa.htm, accessed on 15 May 2020). All chemical reagents were purchased from Sigma-Aldrich (St. Louis, MO, USA).

### 2.8. Micro-CT Scanning and Analysis

Micro-CT scanning was performed after NBF fixation of the skull, lumbar spine, tibia, and femur using Viva-CT 80 (SCANCO Medical AG, Fabrikweg 2, Brüttisellen, Switzerland) with scan parameters of 15 µm voxel size, 70 kVP, and 114 µA to detect HO and characterize bone microarchitecture. Spine L5 trabecular bone was quantified by contouring the entire vertebral trabecular bone while excluding the cortical bone portion using hand contouring and the morph function in the software. Proximal tibia cancellous bone was defined by contouring the trabecular bone right below the growth plate and extended to 50 slices (750 μm total). Both spine L5 and proximal tibia trabecular bone were defined with Gauss Sigma = 0.8, Gauss support = 1, and threshold at 163. The cortical bone microarchitecture of the femur was quantified by defining 50 slices at the midshaft, using Gauss sigma = 0.8, Gauss support = 1, and a threshold of 200, as previously described [9]. To quantify the HO in the surrounding muscle of the femur, we contoured the entire femur surrounding muscle by excluding femur cortical bone, and then defining HO using Gauss Sigma = 0.8, Gauss Support = 1, and a threshold of 163. In this case, total volume (TV) represented total muscle volume and BV represented HO in the muscle. BV/TV represented the ratio of HO volume to total muscle volume.

### 2.9. Bone Histology Analysis and Immunohistochemistry

After micro-CT scans, the bone tissues were decalcified using 10% ethylenediaminetetraacetic acid disodium salt dihydrate (E4884, Sigma-Aldrich, St. Louis, MO, USA) plus 1% sodium hydroxide for 4 weeks. Bone tissues were processed with gradient alcohol, cleared with xylene, and infiltrated in paraffin 9 (Fisher Scientific, Hampton, NH, USA) and paraffin-embedded using the KD-TS3D1 Automatic Tissue Processor and KD-BM & BL Tissue Embedding & Cooling System (Jinhua Kedi Instrumental Equipment. Co. LTD, Jinhua, China). Sections were cut into 5 µm slices using a microtome. Herovici’s staining was performed as previously described [56,57]. H&E staining was performed, as detailed above. TRAP staining was performed using the Leukocyte Acid Phosphatase (TRAP) Kit (387-A Kit, Sigma-Aldrich, St. Louis, MO, USA) following the manufacturer’s protocol. Immunohistochemistry of osterix (OSX) using rabbit anti-OSX (ab 22552, 1:1000, Abcam, Cambridge, UK) was also performed according to the previously described protocol [9]. The secondary antibody used was Goat Anti-Rabbit IgG Antibody (H + L), Biotinylated (BA-1000-1.5, 1:300, Vector Laboratories Inc, Newark, CA, USA). After secondary antibody incubation and washing, slides were incubated with ABC reagents for 2 h at ambient temperature (VECTASTAIN^®^ Elite^®^ ABC-HRP Kit, Peroxidase (Standard) (PK-6100, Vector Laboratories). After another 3 washes with PBS, the DAB Substrate Kit, using Peroxidase (HRP) and Nickel (3,3′-diaminobenzidine) (SK-4100) (Vector Laboratories, Newark, CA, USA), was used to reveal positive cells as brown in color. Slides were washed with tap water and then counterstained with Hematoxylin QS (H-3404-100, Vector Laboratories, Newark, CA, USA) for 30 s and rinsed with tap water. Slides were rinsed with running tap water for 10 min to blue nuclei. Slides were then dehydrated in gradient alcohol, cleared with xylene, and mounted with Cytoseal (Fisher Scientific). Microscopic images were taken with a Nikon Ni bright-field microscope (Nikon Instruments Inc., Melville, NY, USA). The number of TRAP^+^, OC^+^, and OSX^+^ cells was counted using Image J normalized to 1 mm of bone surface.

### 2.10. Statistical Analysis

All data were analyzed using Graphpad Prism 9.4. Student’s *t* tests were used for two group comparisons. Analysis of variance (ANOVA), followed by Tukey’s post hoc multiple comparisons, was used for comparisons of three or more groups. For data without a normal distribution, the Wilcoxon rank sum test was used. *p* < 0.05 was considered statistically significant.

## 3. Results

### 3.1. PGE2/EP2/4 Signaling Pathway Components Were All Significantly Up-Regulated in DKO-Hom Mice

**Figure 1:**
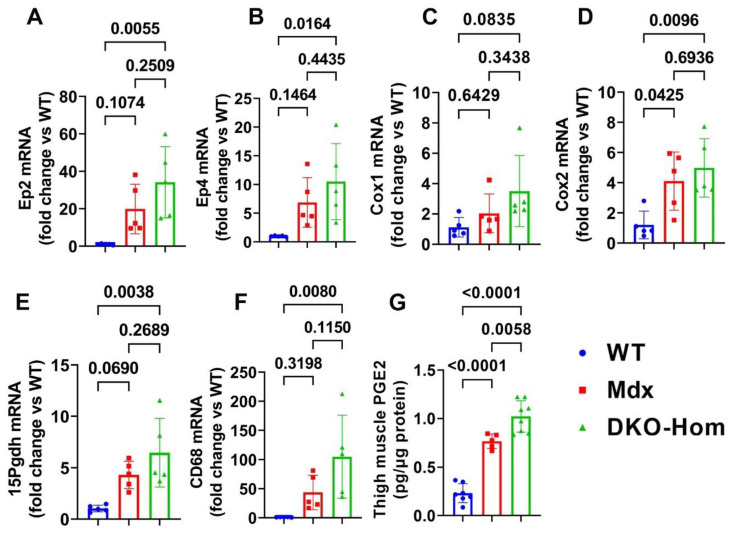
PGE2/EP2/4 pathway mRNA expressions in the thigh muscle of WT, Mdx, and DKO-Hom mice by Q-PCR. (**A**) Ep2 mRNA expression. (**B**) Ep4 mRNA. (**C**) Cox1 mRNA expression. (**D**) Cox2 mRNA expression. (**E**) 15Pgdh mRNA expression. (**F**) CD68 mRNA expression. (**G**) PGE2 level in muscle tissue homogenates of the 4-week-old mice. Exact *p* values are indicated between group bars.

We first performed reverse transcriptive quantitative polymerase chain reaction (Q-PCR) analysis of thigh muscle from wild-type (WT), Mdx, and DKO-Hom mice. We found a trend of increased Ep2 mRNA in Mdx mice compared to WT mice, while Ep2 mRNA increased 30-fold in DKO-Hom mice compared to WT mice (Figure 1A). Ep4 mRNA also significantly increased in Mdx mice but increased 10-fold in DKO-Hom mice thigh muscle compared to WT mice (Figure 1B). Cyclooxygenase 1 (Cox-1) mRNA expression in the thigh muscle of Mdx mice showed no significant change compared to WT mice but demonstrated an increasing trend in DKO-Hom mice (*p* = 0.0835) (Figure 1C). Cyclooxygenase 2 (Cox-2) mRNA expression significantly increased in both Mdx and DKO-Hom mice compared to WT mice (*p* = 0.045 and 0.0096, respectively) (Figure 1D). 15-hydroxyprostaglandin dehydrogenase (15-Pgdh), the enzyme that catabolizes the degradation of PGE2, also increased in Mdx mice and significantly increased by 6-fold in DKO-Hom mice compared to WT mice (Figure 1E). Further, cell differentiation 68 (CD68), an M1 macrophage marker, significantly increased in the thigh muscle of DKO-Hom mice compared to the WT mice **(**Figure 1F). ELISA results showed that PGE2 was significantly elevated in the thigh muscle tissues of Mdx and DKO-Hom mice. PGE2 was also significantly higher in DKO-Hom mice compared to Mdx mice (Figure 1G).

### 3.2. Increased Osteogenic Genes Correlated with HO in the Muscle Tissues of DKO-Hom Mice

**Figure 2:**
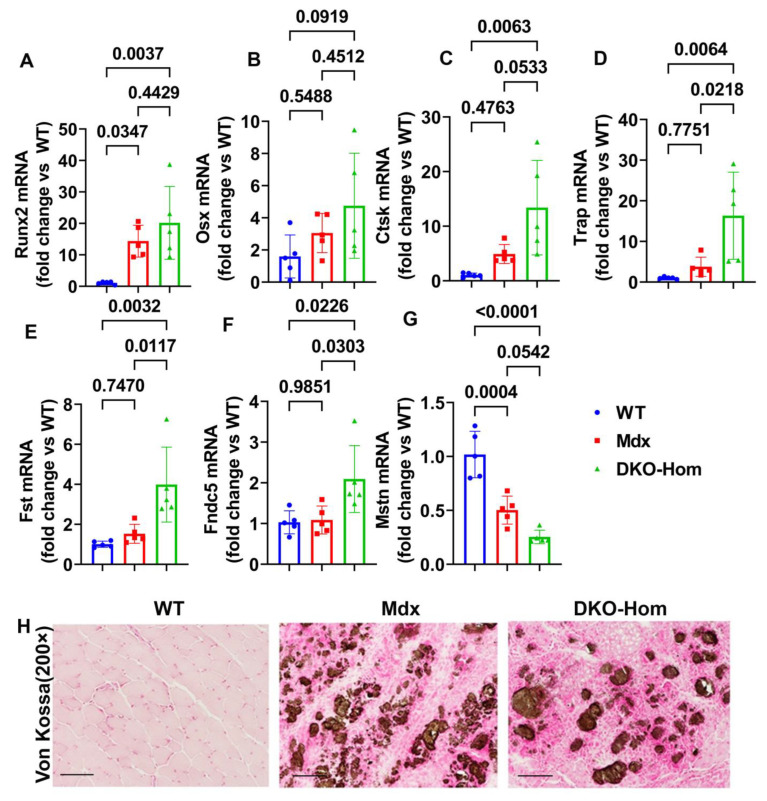
Osteogenesis, osteoclastogenesis, and myokine mRNA expression in 4-week-old mice and von Kossa staining. (**A**) Runx2 mRNA expression. (**B**) Osx mRNA expression. (**C**) Ctsk mRNA expression. (**D**) Trap mRNA expression. (**E**) Fst mRNA expression. (**F**) Fndc5 mRNA expression. (**G**) Mstn mRNA expression. (**H**) Von Kossa staining for gastrocnemius muscle tissues. Significant brown-black color staining was found in the gastrocnemius muscle of Mdx and DKO-Hom mice, which indicates heterotopic bone formation (mineralization). Scale bar = 100 µm. Exact *p* values are indicated between group bars.

Since we previously demonstrated that both Mdx and DKO-Hom muscle have severe HO formation, as revealed by micro-CT [9], we further tested osteogenesis and osteoclastogenesis gene expression in the thigh muscle of Mdx and DKO-Hom mice. The Q-PCR results showed that Runt-related transcription factor 2 (Runx2) was significantly increased in both Mdx and DKO-Hom mice compared to WT mice (*p* = 0.0347 and 0.0037, respectively) (Figure 2A). Osterix (Osx) mRNA also showed an increasing trend in DKO-Hom mice when compared to WT control mice (*p* = 0.0919) (Figure 2B). Furthermore, cathepsin K (Ctsk) was found to be significantly elevated in DKO-Hom mice when compared to Mdx and WT mice, while no significant increases were observed in Mdx mice compared to WT mice (Figure 2C). Further, tartrate-resistant acid phosphatase (Trap) mRNA expression was also significantly increased in DKO-Hom mice compared to Mdx and WT mice (*p* = 0.0218 and 0.0064, respectively) (Figure 2D). These results indicate that osteogenesis and osteoclastogenesis were both active in the muscle tissues of DKO-Hom mice and likely contributed to HO. We further investigated the myokine expression in the thigh muscle tissues. We found that follistatin (Fst) was significantly increased in the DKO-Hom mice compared to the Mdx and WT mice (*p* = 0.0117 and 0.0032, respectively) (Figure 2E). In addition, irisin (Fndc5) was also significantly up-regulated in the DKO-Hom mice compared to the Mdx and WT mice (*p* = 0.0303 and 0.0226, respectively) (Figure 2F). In addition, myostatin (Mstn) was significantly decreased in Mdx and DKO-Hom mice compared to WT mice (*p* = 0.0004 and <0.0001, respectively) (Figure 2G). These myokine changes favor muscle regeneration and likely compensate for the muscle damage due to the lack of dystrophin and utrophin. Finally, von Kossa staining of the gastrocnemius muscle tissues revealed brown-black mineralization in the muscle tissues of both the Mdx and DKO-Hom mice, which further proved HO formation (Figure 2H).

### 3.3. EP2/EP4 Expression in the Muscle Tissues Was Predominately in the Macrophages

**Figure 3:**
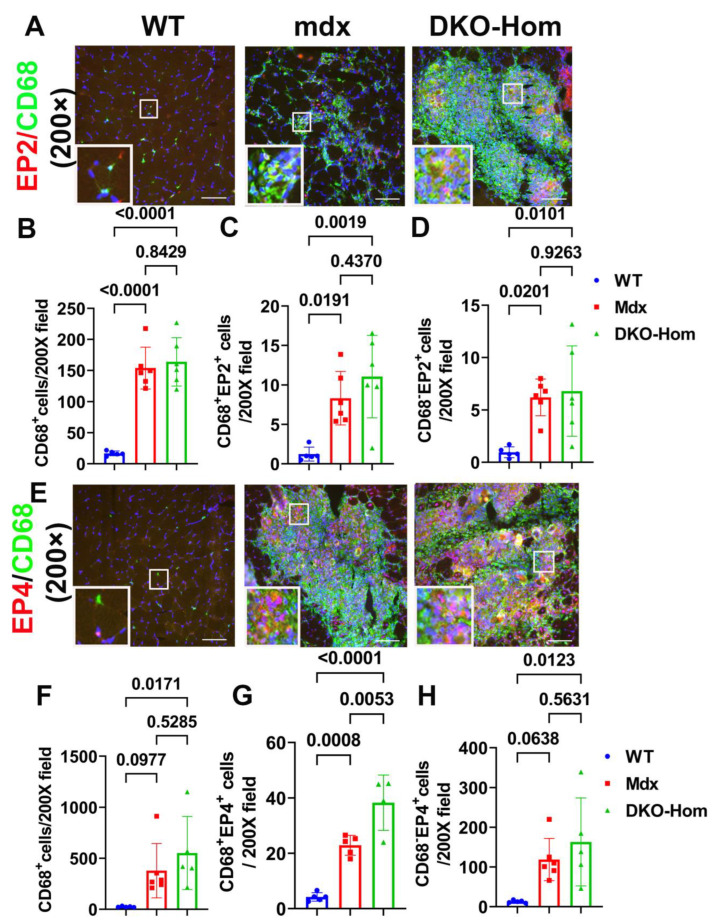
Immunofluorescence staining of EP2 and EP4 receptors and colocalization with CD68 macrophages in 4-week-old muscle tissues. (**A**) EP2/CD68 double staining. CD68 stained with green, EP2 stained red, colocalized cells showed yellow or orange color. (**B**–**D**) Quantification of CD68^+^macrophages and CD68^+^EP2^+^ and CD68^−^EP2^+^ cells. (**E**) EP4/CD68 double staining. EP4 stained red, CD68 stained green. Colocalized cells stained yellow or orange. (**F**–**H**) Quantification of CD68^+^ macrophages and CD68^+^EP4^+^ and EP4^+^ cells. Exact *p* values are indicated between group bars. Scale bars = 100 µm.

Next, we performed immunofluorescence staining for the gastrocnemius muscle tissues to detect which cells expressed EP2/EP4. We colocalized EP2 with CD68, an M1 macrophage marker. We found a significant increase in CD68^+^ macrophages in Mdx and DKO-Hom mice compared to WT control mice (*p* < 0.0001 for both) (Figure 3A,B). CD68^+^ EP2^+^ double-positive cells were also significantly increased in Mdx and DKO-Hom mice as compared to WT mice (*p* = 0.0191 and 0.0019, respectively) (Figure 3A,C). There was also a significant increase in CD68^−^ EP2^+^ cells in the muscle tissues of Mdx and DKO-Hom mice (Figure 3A,D). These results indicate that EP2 expression was significantly increased in both macrophages and non-macrophages in the muscle tissues of Mdx and DKO-Hom mice. CD68 and EP4 double staining was performed and revealed a significant increase in CD68^+^ cells in the muscle tissues of DKO-Hom mice compared to WT mice (*p* = 0.0171) (Figure 3E,F). Furthermore, CD68^+^EP4^+^ cells were also significantly elevated in the muscle tissues of both Mdx and DKO-Hom mice compared to WT mice (*p* = 0.0008 and <0.0001, respectively) (Figure 3E,G). CD68^−^EP4^+^ cells were also significantly increased in the DKO-Hom mice (*p* = 0.0123) (Figure 3E,H). These results demonstrate that EP4 was significantly increased in both macrophages and non-macrophages in the gastrocnemius muscle tissues of DKO-Hom mice.

### 3.4. Treatment with EP2 Antagonist PF04418948 Decreased HO in the Muscle Tissues

**Figure 4:**
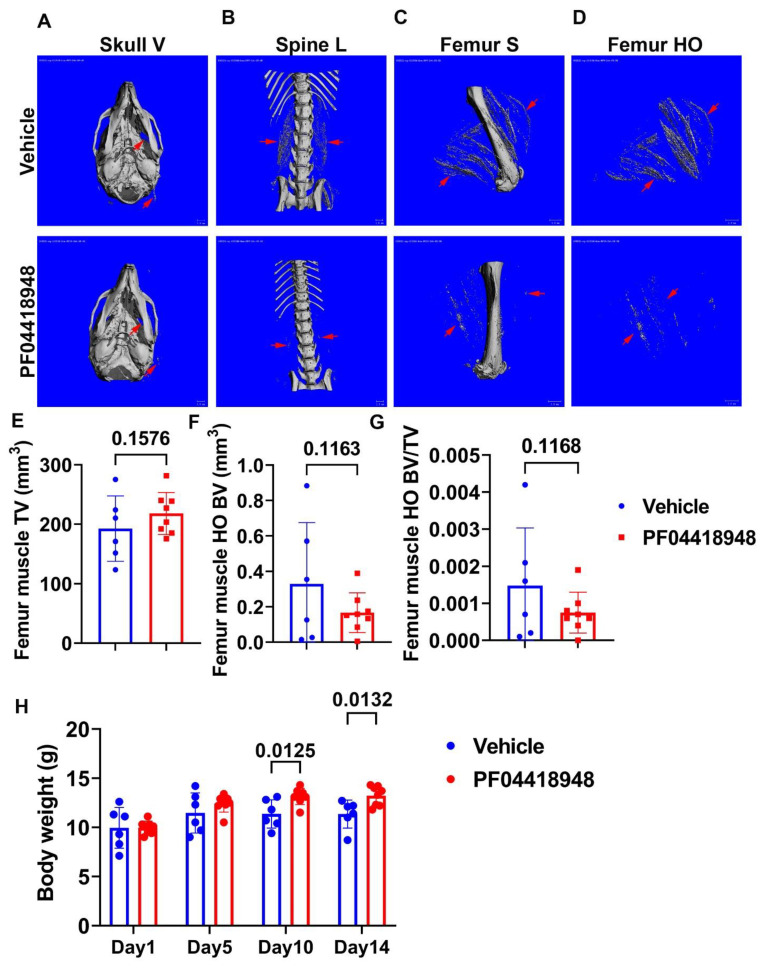
Effect of EP2 antagonist PF04418948 on muscle HO formation. (**A**) Micro-CT 3D images of the entire skull. (**B**) Lumbar spine overview for HO formation in spine surrounding muscles. (**C**) Micro-CT sagittal view of femur surrounding muscle tissues. (**D**) Micro-CT 3D segmental view of HO in thigh muscles. Red arrows indicate HO in all images. (**E**) Quantification of TV of the entire thigh muscle. (**F**) Micro-CT BV of the entire thigh muscle. (**G**) BV/TV of HO in the thigh muscle. (**H**) Body weight at different time points of treatment. Scale bars for micro-CT images = 1 mm.

To further investigate the role of the EP2/PGE2 signaling pathway in HO formation in muscle, we treated DKO-Hom mice at 4 weeks old with the EP2 antagonist PF04418948 for 2 weeks by oral gavage at the dose of 10 mg/kg/day. Mice were sacrificed 2 weeks after PF04418948 treatment. Micro-Computer Tomography (Micro-CT) overviews of the skull (ventral view, Skull-V) (Figure 4A), lumbar spine (ventral view, Spine L) (Figure 4B), and femur (sagittal view, Femur-V) (Figure 4C) showed an obvious decrease in HO formation in the muscle tissues in PF04418948-treated mice compared to the vehicle-treated mice. To quantify HO, we segmentalized the entire femur muscle HO by drawing contours to exclude bone tissues. The segmental HO formation showed obvious decreases in the PF04418948-treated group compared to the vehicle-treated group (Figure 4D). Quantification of the total muscle volume (TV) showed a increasing trend in PF04418948-treated mice compared to vehicle-treated mice (*p* = 0.1576) (Figure 4E). Total femur muscle HO volume (BV) showed a decreasing trend (*p* = 0.1163) in PF04418948-treated versus vehicle-treated muscle (Figure 4F). Furthermore, BV/TV also tended to decrease in the PF04418948-treated mice compared to the vehicle-treated mice (*p* = 0.1168) (Figure 4G). Additionally, mouse body weight was also significantly higher in the PF04418948-treated group compared to the vehicle-treated group at days 10 and 14 after treatment (Figure 4H).

### 3.5. PF04418948 Treatment Improved Muscle Pathology

**Figure 5:**
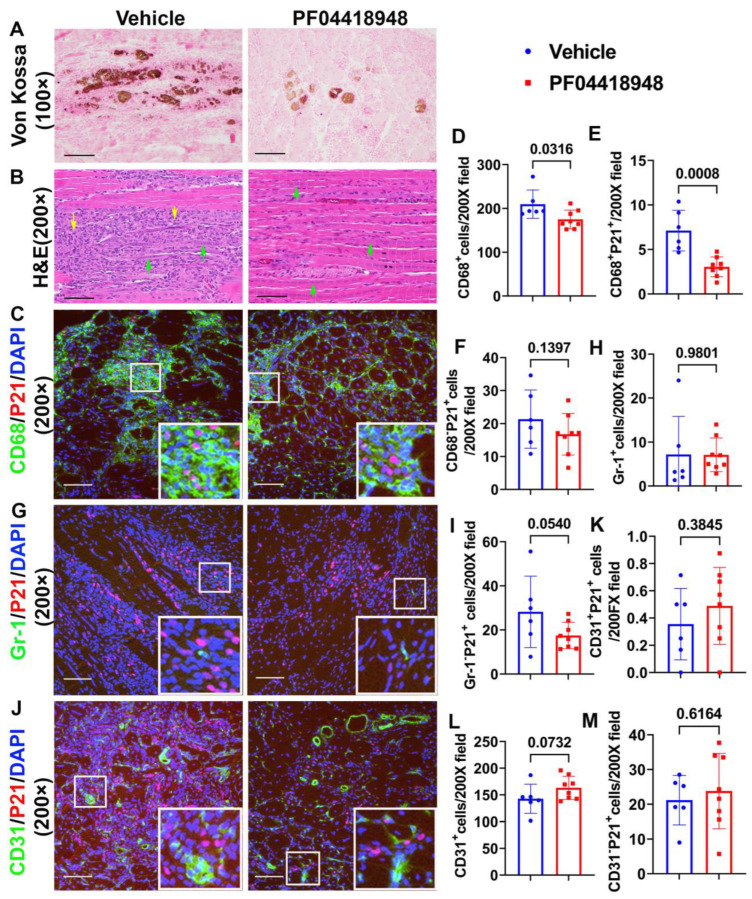
Muscle histology and inflammation after treatment with PF04418948. (**A**) Von Kossa staining for HO. Brown-black color indicates HO. Scale bar = 200 µm. (**B**) H&E staining. A large number of inflammatory cells were identified in the vehicle-treated group, while more regenerated muscle fibers were identified in the PF04418948 group. Yellow arrows indicate inflammatory cells, green arrows indicate regenerating multiple nucleated cells. Scale bars = 100 µm. (**C**) CD68/P21 double staining. Insets highlight positive cells, with CD68 stained green and P21 stained red. Scale bars = 100 µm. (**D**–**F**) Quantification of CD68^+^, CD68^+^P21^+^, and CD68^−^P21^+^cells. (**G**) Gr-1/P21 double staining. Insets highlight positive cells, with Gr-1 stained green and P21 stained red. Scale bars = 100 µm. (**H**,**I**) Gr-1^+^ and Gr-1^−^P21^+^ cell quantification. (**J**) CD31/P21 double staining Insets highlight positive cells. CD31 stained with green, P21 stained with red. Scale bars = 100 µm. (**K**–**M**) CD31^+^P21^+^, total CD31^+^ and CD31^−^P21^+^ cell quantification. Exact *p* values are shown between group bars.

To further investigate if PF04418948 treatment improves muscle pathology, von Kossa staining was used to detect HO formation. The overall results showed that PF04418948 treatment decreased the von Kossa-positive area, a finding consistent with the results from the micro-CT (Figure 5A). Furthermore, Hematoxylin and Eosin (H&E) staining demonstrated a large number of inflammatory cell patches (yellow arrows) in the vehicle-treated group, while muscle from the PF04418948-treated group showed decreased inflammation and increased regeneration of long myofibers (multiple nucleated cells, green arrows) (Figure 5B). To investigate if PF04418948 treatment decreased inflammation and senescent cells, double immunofluorescent staining of CD68 (M1 macrophage) and P21 (senescent cells marker) was performed and revealed fewer green-stained macrophages in the PF04418948-treated group compared to the vehicle-treated group. Furthermore, more central nuclear myofibers were identified in the PF04418948-treated group (Figure 5C). Quantification indicated that total CD68^+^ cells/200 × field and CD68^+^P21^+^ senescent macrophages were significantly decreased in the PF04418948-treated group compared to the vehicle-treated group (*p* = 0.0316 and 0.0008, respectively) (Figure 5D,E). PF04418948 treatment also showed a decreasing trend in CD68^−^P21^+^ (non-macrophage) senescent cells (*p* = 0.1397) (Figure 5F). We also performed Gr-1 (neutrophil) and P21 double immunofluorescent staining, which revealed that PF04418948 treatment did not change the total Gr-1^+^positive cells, which was very low (Figure 5H). However, Gr-1^−^P21^+^ cells (non-neutrophil senescent cells) were reduced by PF04418948 treatment (Figure 5I).

Finally, we performed CD31/P21 double immunofluorescence staining, which revealed no significant differences in CD31^+^P21^+^ cells (senescent endothelial cells) between the PF04418948-treated and vehicle-treated groups (Figure 5J,K). A trend of increased total CD31^+^ cells was detected in the PF04418948-treated group (Figure 5I,L), but no differences were detected in CD31^−^P21^+^ cells between the PF04418948-treated group and the control groups (Figure 5M).

### 3.6. PF04418948 Treatment Changed HO-Related Genes, Myokine, and Inflammatory Genes

**Figure 6:**
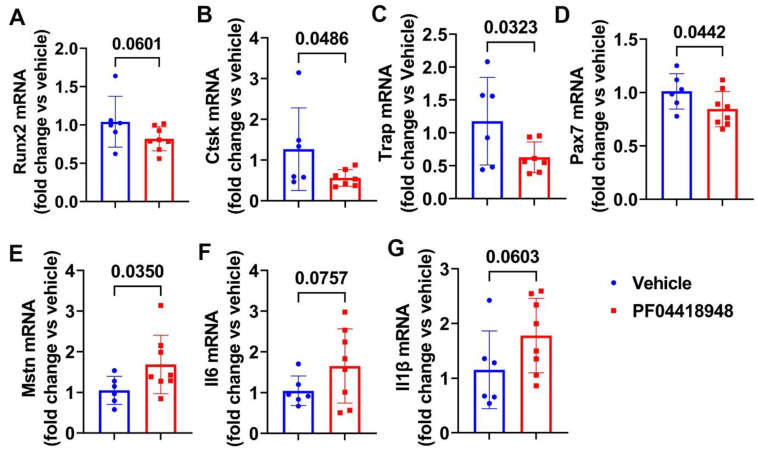
Muscle gene expression after treatment with PF04418948 detected by Q-PCR. (**A**) Runx2 mRNA expression. (**B**) Ctsk mRNA expression. (**C**) Trap mRNA expression. (**D**) Pax-7 mRNA expression. (**E**) Mstn mRNA expression. (**F**) Il6 mRNA expression. (**G**) Il-1β mRNA expression.

We further performed Q-PCR analysis of the thigh muscle tissues for PF04418948 and the vehicle-treated mice. We found a decreasing trend of Runx2 mRNA expression in the PF04418948-treated group compared to the vehicle-treated group (Figure 6A). PF04418948 treatment significantly decreased Ctsk and Trap mRNA expression compared to the vehicle treatment (Figure 6B,C). Furthermore, PF04418948 treatment also significantly decreased Pax-7 expression (Figure 6D) and increased Mstn expression (Figure 6E). However, PF04418948 treatment demonstrated an increasing trend of IL-6 and IL1-β mRNA expression compared to the vehicle-treated group (*p* = 0.0767 and 0.0603, respectively).

### 3.7. PF04418948 Treatment Improved the Trabecular Bone and Cortical Bone Microarchitecture of Long Bones

**Figure 7:**
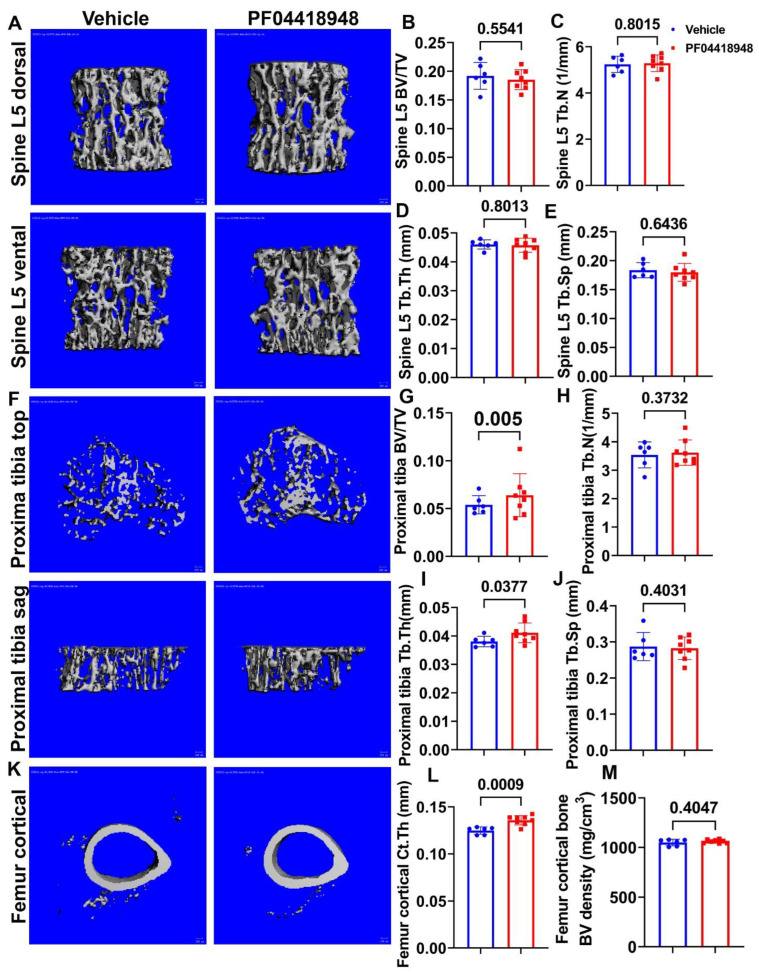
Micro-CT analysis of bone tissues. (**A**) Micro-CT 3D images of spine L5 with ventral and dorsal views. (**B**–**E**) BV/TV, Tb.N, Tb.Th, and Tb.Sp of spine L5. (**F**) Micro-CT 3D images of top and sagittal views of the proximal tibia trabecular bone. (**G**–**J**) BV/TV, Tb.N, Tb.Th, and Tb.Sp of proximal tibia trabecular bone. (**K**) Micro-CT 3D view of femur cortical bone. (**L**,**M**) Ct.Th and BV density of femur cortical bone. Scale bars = 100 µm. For proximal tibia BV/TV, Wilcoxon rank sum test was used due to the data not having a normal distribution. All other comparisons used Student’s *t* test.

Micro-CT 3D images and quantification demonstrated that PF04418948 treatment did not change any trabecular bone parameters of the spine L5 vertebrate trabecular bone, including BV/TV, trabecular number (Tb.N), trabecular thickness (Tb.Th), and trabecular separation (Tb.Sp) (Figure 7A–E). Furthermore, Micro-CT 3D images and quantification revealed that PF04418948 treatment significantly increased BV/TV and Tb.Th and demonstrated an increasing trend of BV density (613.74 vs. 559.28 mgHA/cm^3^, *p* = 0.116) in the proximal tibia trabecular bone compared to the vehicle-treated group. No differences were found for Tb.N or Tb.Sp in the proximal tibia trabecular bone (Figure 7F–J). PF04418948 treatment also significantly increased the cortical thickness (Ct.Th) of femur cortical bone (Figure 7K,L), while not changing BV density (Figure 7M). In addition, PF04418948 treatment led to an increasing trend of tibia Ct.Th (0.1362 mm vs. 0.1303 mm, *p* = 0.07) when compared to the vehicle-treated group and did not change the BV density of tibia cortical bone (1052.2276 vs. 1052.7739 mgHA/cm^3^). These results, taken together, demonstrate that PF04418948 treatment improved the microarchitecture of long bones, while not impacting spine trabecular bone.

### 3.8. Effects of PF04418948 Treatment on General Bone Morphology and Bone Matrix Collagen Type 1 (COL1)

**Figure 8:**
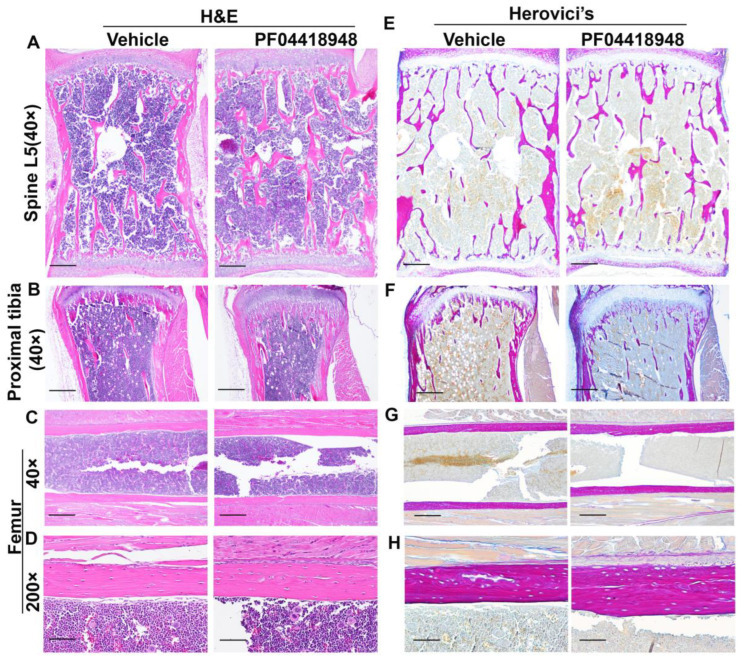
H&E staining and Herovici’s staining of bone tissues of DKO-Hom mice treated with PF04418948 or vehicle. (**A**) H&E staining of spine L5 trabecular bone. No significant difference between PF04418948-treated and vehicle-treated group was identified. (**B**) H&E staining of proximal tibia. More trabecular bone extended to the distal side of the proximal tibia in the PF04418948-treated group compared to the vehicle-treated group. (**C**,**D**) H&E staining of femur cortical bone at 40× and 200×. PF04418948-treated group showed thicker femur cortical bone than vehicle-treated group. (**E**) Herovici’s staining of spine L5 trabecular bone. COL1 stained a pink-red color, COL3 stained a dark blue color. No difference was found between the PF04418948-treated group and vehicle-treated group. (**F**) Herovici’s staining of proximal tibia. The PF04418948-treated group showed more trabecular bone than the vehicle-treated group, extending to the distal side of the proximal tibia. (**G**,**H**). Herovici’s staining of femur cortical bone at 40× and 200×. Femur cortical collagen 1 (pink-red)-positive matrix was thicker in the PF04418948-treated than the vehicle-treated group. Scale bars = 500 µm for 40× and 100 µm for 200×.

H&E staining of the spine L5 vertebrate revealed no significant difference in the trabecular bone structure between the PF04418948-treated and vehicle-treated groups (Figure 8A). Relatively more trabecular bone was found in the proximal tibia of the PF04418948-treated group than the vehicle-treated group and extended to the distal end of the proximal tibia (Figure 8B). However, H&E staining of femoral cortical bone showed increased thickness in the PF04418948 group compared to the vehicle-treated group (Figure 8C,D). Herovici’s staining, which differentiates COL 1 (pink-red) and collagen type 3 (COL3) (dark blue), stained the spine L5 vertebrate trabecular bone with a pink-red color and bone marrow with a light-blue color. No differences in trabecular bone number or thickness were found between the PF04418948-treated group and the vehicle-treated group (Figure 8E). Herovici’s staining of the proximal tibia demonstrated more pink-red collagen 1-positive trabecular bone in the PF04418948-treated group, extending to the distal side of the proximal tibia, compared to the vehicle-treated group (Figure 8E). More strikingly, in the femur cortical bone, pink-red COL1 was obviously thicker in the PF04418948-treated group than in the vehicle-treated group (Figure 8G,H).

### 3.9. PF04418948 Treatment Increased Osteoclasts Without Changing the Number of Osteoprogenitors in the Trabecular Bone of the Proximal Tibia

**Figure 9:**
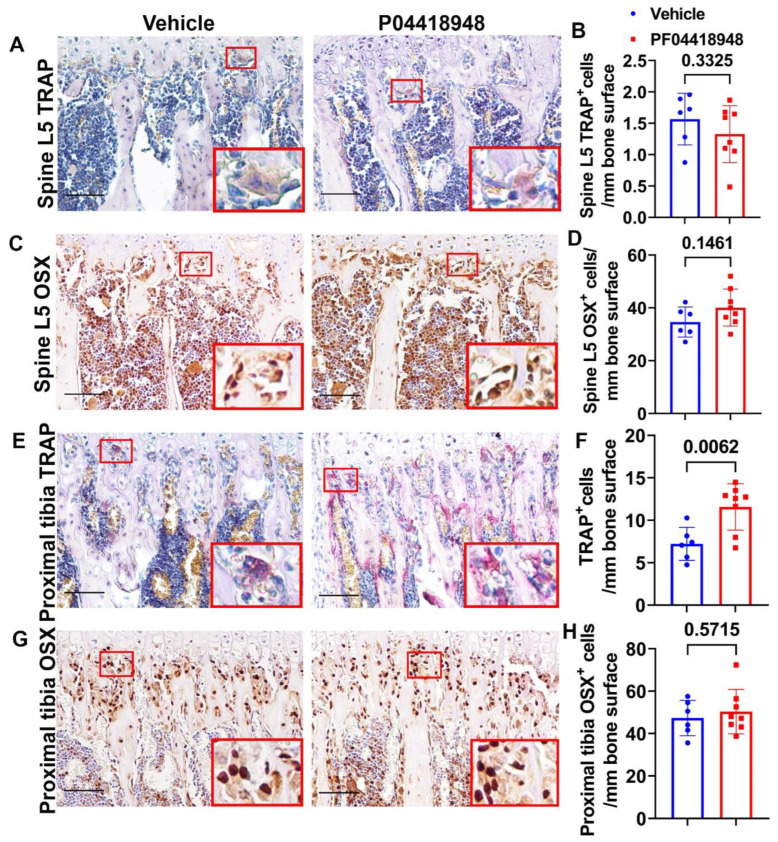
Bone osteoclasts and osteoblast changes after PF04418948 treatment for DKO-Hom mice. (**A**,**B**) TRAP staining of spine L5 vertebrate trabecular bone. TRAP^+^ osteoclasts were stained violet-red. No significant difference for TRAP^+^ cells/bone surface between the PF04418948- and the vehicle-treated groups. (**C**,**D**) Immunohistochemistry staining of OSX for spine L5 vertebrate trabecular bone. OSX^+^ cells were stained a brown color in the nuclei. OSX^+^-positive cells/bone surface showed an increasing trend in the PF04418948-treated group. *p* = 0.1461. (**E**,**F**) TRAP staining of proximal tibia and quantification. PF04418948 treatment significantly increased TRAP^+^ cells compared to the vehicle-treated group. (**G**,**H**) Immunohistochemistry staining of OSX of the proximal tibia. No difference between PF04418948-treated and the vehicle-treated mice. Scale bars = 100 µm for all the images.

TRAP staining for osteoclasts was performed and quantified for the spine L5 trabecular bone. No significant differences in TRAP^+^ cells were observed on the bone surface for the spine trabecular bone in the PF04418948-treated group when compared to the vehicle-treated group (Figure 9A,B). Immunohistochemistry staining of OSX for osteoprogenitor cells showed a trend of increasing OSX^+^ cells (*p* = 0.1461) in the PF04418948-treated group compared to the vehicle-treated group (Figure 9C,D). For the proximal tibia trabecular bone, PF04418948 treatment significantly increased TRAP^+^ osteoclasts compared with the vehicle-treated group (Figure 9E,F). However, PF004418948 treatment did not significantly increase the OSX^+^ osteoprogenitor cells compared to the vehicle-treated group (Figure 9G,H).

## 4. Discussion

**Figure 10:**
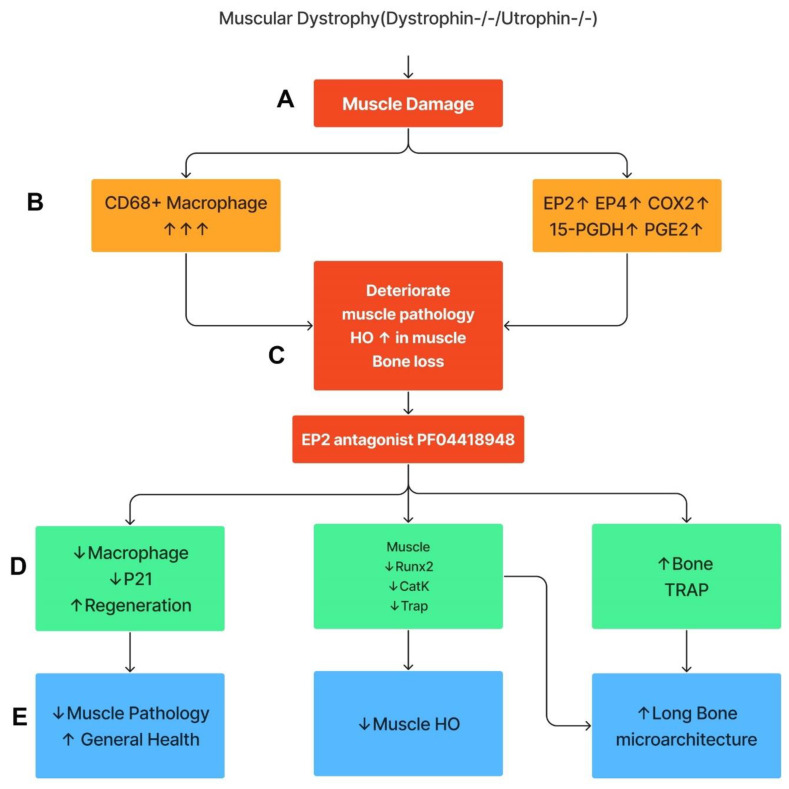
Schematic mechanism summary of the results. This graph was created by Xiang Xiao using Figma. (**A**) DKO-Hom mice developed severe muscle inflammation due to loss of functional dystrophin and compensation of Utrophin, which caused muscle fiber necrosis and subsequent macrophage infiltration. (**B**) Macrophages expressed high levels of COX-2/PGE2/EP2/4 signaling pathway molecules. (**C**) Persistent macrophage infiltration and activation of COX2-signaling pathway deteriorated muscle pathology and increased HO formation, resulting in bone loss. (**D**) Treatment of mice with the EP2 antagonist decreased senescent macrophages, increased muscle regeneration via decreases in Runx2, CatK, and TRAP, and increased bone osteoclastogenesis. (**E**) PF04418948 treatment further decreased muscle HO and muscle pathology and improved bone growth and microarchitecture. Upward arrows indicate increased changes. Downward arrows indicate decreased changes.

This study demonstrates that the components of COX2/PGE2/EP2/4 are all significantly up-regulated in Mdx mice, with more striking elevations in DKO-Hom mice compared to WT mice. Although 15-PGDH, the enzyme which degrades PGE2, was also increased, the net result was a significant elevation in PGE2 in the muscle tissues of the DKO-Hom mice. Both EP2 and EP4 were expressed in both CD68^+^macrophage and non-macrophage cells. PF04418948 treatment improved muscle pathology and decreased HO formation at a 10 mg/kg dosage, potentially by decreasing the total CD68^+^ macrophages and senescent macrophages (CD68^+^P21^+^) without affecting neutrophils, while increasing CD31^+^ endothelial cells. PF04418948 treatment also increased tibia trabecular bone BV/TV, Tb.Th, and femoral and tibia Ct.Th without negatively affecting the trabecular bone microarchitecture of spine L5. Our major findings are summarized in Figure 10.

Inflammation is one of the most profound clinical manifestations of DMD. Glucocorticoids (Deflazacort or prednisone) are currently considered the gold-standard care for muscular dystrophy, improving the overall health of DMD patients and extending ambulatory time [58,59,60]. Further, Deflazacort-treated boys have a longer duration of ambulation than prednisone-treated patients (15.6 vs. 13.5 years). Furthermore, Deflazacort was also associated with a lower risk of scoliosis, improved ambulatory function, a greater percentage of lean body mass, shorter stature, and lower body weight, after adjusting for age and steroid duration [61]. However, glucocorticoid treatment, including Deflazacort, increased vertebrate fracture risk for patients with DMD (with a reported 16-fold increase for some study), and boys with a history of fracture(s) had a steep decline in function [33,34,62,63]. Therefore, the development of approaches targeting inflammation without negatively affecting bone is needed, in addition to using gene therapy to restore dystrophin.

This study first thoroughly investigated the role of the PGE2-EP2/4 pathway in the pathogenesis of DKO-Hom mice with the goal of developing a potential therapy for the treatment of DMD patients. The Q-PCR results showed that Cox2, Ep2, and Ep4 as well as 15-Pgdh are all significantly increased in the thigh muscle tissues of both Mdx and DKO-Hom mice, while COX1 was not significantly changed. Furthermore, the ELISA results demonstrated a significant elevation in PGE2 in both Mdx and DKO-Hom mice, although its degradation enzyme, 15-Pgdh, was also significantly increased. CD68 (M1 macrophage marker) mRNA was also dramatically increased in DKO-Hom mice (Figure 1). Immunofluorescence staining demonstrated that EP2 and EP4 receptors were present at significantly higher levels, in both macrophages and non-macrophages, in DKO-Hom mice compared to WT mice (Figure 2).

Thus far, very few studies have investigated PGE2 pathways in muscular dystrophy. Previous studies showed that Mdx mice muscle tissues released more PGE2 in response to contraction [50,64] and followed a similar pattern of creatine kinase release. In myotonic dystrophy type 1 (DM1), PGE2 was found to be increased through the up-regulation of Cox2, microsomal prostaglandin E synthase-1 (mPGES-1), and prostaglandin EP2/EP4 receptors. The up-regulation of PGE2 suppressed myogenic differentiation by decreasing the intracellular levels of calcium. The exogenous addition of acetylsalicylic acid, an inhibitor of Cox enzymes, abolished abnormal PGE2 secretion and restored the differentiation of DM1 muscle cells [65]. PGE2/EP2 acted downstream of Notch signaling by inhibiting myogenic differentiation and promoting the self-renewal of human muscle progenitor cells [49]. Other study also demonstrated that COX2-KO muscle-derived stem cells are more easily differentiated into myofibers in calvarial bone defects, even when they were transduced with retro-viral BMP4 [66]. However, no study has investigated PGE2 pathways in the DKO-Hom mice, which closely mimic the manifestations of DMD patients.

Since HO is one of the major muscle pathology changes in DKO-Hom mice [9,67], the osteogenic genes were further investigated, and it was found that Runx2, Ctsk, and Trap mRNA were all significantly increased in Mdx mice and DKO-Hom mice, while Osx also showed a trend of up-regulation in DKO-Hom mice compared to WT mice. These results indicate that osteogenic genes and osteoclastogenic genes are up-regulated in the muscle tissues of DKO-Hom mice, likely due to HO formation. A recent study showed that human muscle from DMD patients also exhibited a 16-fold increase in Ctsk when compared to normal muscle [68]. It is likely that osteoclasts exist in the human muscles of DMD patients due to HO formation. However, Fst and Fndc5 (irisin) were significantly increased, while Mstn was significantly decreased in Mdx and DKO-Hom mice compared to WT mice (Figure 2). These myokine gene expression changes favor muscle regeneration and likely did not contribute to the functional decline of DKO-Hom mice.

To further elucidate the role of PGE2/EP2 in the development of muscle pathology and HO formation, we treated DKO-Hom mice at 4 weeks old (a time when significant muscle damage and osteopenia are observed) with the EP2 antagonist PF04418948 for two weeks. Treatment with PF04418948 decreased HO, as demonstrated by micro-CT (skull, spine, and long bone surrounding muscle), and showed a decreasing trend of BV and BV/TV in the thigh muscle tissues. Von Kossa staining also revealed decreased HO formation in the muscle tissues. H&E staining showed improved muscle pathology, as revealed by a decrease in inflammatory cells, with increased regenerated myofibers. PF04418948 treatment decreased total macrophages (CD68^+^) and senescent macrophages (CD68^+^/P21^+^) but did not change the number of Gr-1^+^ cells or Gr-1^+^/P21^+^ cells, which was very low, and increased total CD31^+^ cells with no changes in CD31^+^/P21^+^ cells, which were also very sparse in number (Figure 5). The Q-PCR results further showed that Runx2, Ctsk, and Trap expression were down-regulated, which likely correlated with the decreased formation of HO as demonstrated by micro-CT and von Kossa staining. Interestingly, Pax7 was down-regulated, while Mstn expression was up-regulated following treatment with PF04418948. Previously, it has been reported that Pax7 and myogenin expression are mutually exclusive during myogenic differentiation; Pax7 appears to be up-regulated in cells that escape differentiation and exit the cell cycle, suggesting a regulatory relationship between these two transcription factors. Indeed, overexpression of Pax7 down-regulates MyoD, prevents myogenin induction, and blocks MyoD-induced myogenic conversion of 10T1/2 cells. Overexpression of Pax7 also promotes cell cycle exit, even under proliferation conditions [69]. Pax7 is also highly expressed during early myogenic differentiation of iPSCs and down-regulated when myogenin is induced [70]. A previous study showed Pax7 is increased in the muscle tissues at 1 and 4 weeks in DKO-Hom mice and is decreased at 8 weeks compared to Mdx mice [5], while showing no difference compared to WT and Mdx mice at 4 weeks and a decrease compared to WT and Mdx mice at 8 weeks [4]. The changes observed in the PF04428948-treated group are likely due to decreased muscle inflammation and increased regenerating fibers differentiated from the PAX7 ^+^ satellite cells. The increase in Mstn is likely due to the improvement of muscle pathology, and its expression returns to a normal level because DKO-Hom mice have significantly decreased Mstn (Figure 2). The observation of no significant changes in Fst and Fndc5 indicates that the improvement of the muscle pathology was not due to the regulation of these positive myokines. The insignificant changes in IL6 and IL1-β indicate that the beneficial effects of PF04418948 are not mediated by the regulation of IL16 and IL1-β. It has been shown that RhoA activation in macrophages also contributes to muscle HO formation [67] and that RhoA/Rock inhibition improves the beneficial effects of glucocorticoids in DKO-Hom mice [71]. The current study has discovered a new mechanism of HO formation in DKO-Hom mice.

Previously, we have shown that DKO-Hom mice develop osteopenia at 4 weeks of age [9]. Importantly, in this study, we demonstrated that PF04418948 treatment increased the BV/TV and Tb.Th of the proximal tibia. Strikingly, PF04418948 treatment increased the Ct.Th of the midshaft femur in addition to producing a trend of increasing tibia cortical bone thickness. This was further verified by COL1 staining (Herovici’s staining) (Figure 8). Increased TRAP^+^ osteoclasts were found in the proximal tibia, though no changes in the OSX^+^ osteogenic progenitor cells of the tibia trabecular bone were observed. Our previous study demonstrated that osteoblasts, osteoclasts, and osteocytes were all decreased in 4-week-old DKO-Hom mice trabecular bone [9], but that osteoclasts increased at 6 weeks [9]. The increased number of osteoclasts in the proximal tibia was also likely caused by decreased inflammation (macrophages) in the muscle, because monocyte–macrophages are also the progenitor cells of osteomacs, which differentiate into osteoclasts on the bone surface [72,73]. When muscle inflammation is improved, more monocyte–macrophage lineage cells will undergo osteoclast differentiation and resume the normal balance of osteogenesis and osteoclastogenesis in the bone tissues.

A previous study demonstrated that PGE2 increased during HO formation from day 2 to day 21, with the peak level reached at day 10 at 33-fold compared to the control in the rabbit model, which was induced by the daily forcible flexion of immobilized knees. Use of the PGE2 receptor antagonist, AH 6809, and blocking cAMP with Rp-cAMP prevented HO formation [74]. Another study created an HO model using quadriceps muscle injury and the transplantation of bone marrow cells. It was found that non-selective COX1 and COX2 inhibitors or selective COX2 inhibitors could rapidly decrease PGE2 levels and inhibit HO formation, which indicates that COX2, rather than COX1, plays an important role in traumatic HO formation [75]. Therefore, our finding that EP2 antagonist inhibit HO formation, while also improving the bone microarchitecture of long bones, may also have implications for traumatic HO treatment, which needs further study.

The limitation of this study is that the treatment duration was only 2 weeks due to the rapid decline in the overall health of the DKO-Hom mice. Whether long-term treatment would further improve muscle pathology and maintain bone microarchitecture requires additional studies. A previous study showed that COX2KO mice have delayed fracture healing and can be largely rescued by an EP4 agonist, while EP2 agonists only marginally rescue the fracture healing phenotype of COX2KO mice. These results indicate that EP2 plays a less important role in PGE2-mediated bone formation [76]. Therefore, targeting EP2 using antagonists may be relatively safer for bone homeostasis. Periodic treatment may be more optimal, without the side effects observed in corticoid treatment.

## 5. Conclusions

In summary, this study elucidated the role of the PGE2/EP2 pathway in the development of muscle and bone pathology in a DKO-Hom mouse model. The EP2 antagonist PF04418948 improved muscle histopathology and the overall health of the DKO-Hom mice. PF044418948 treatment also increased proximal tibia trabecular bone BV/TV and Tb.Th, while increasing femoral and tibial cortical bone thickness without affecting the spine L5 trabecular bone microarchitecture. Therefore, targeting EP2 could be a potential palliative therapy for the treatment of DMD.

## Figures and Tables

**Table 1:** Primer information.

Gene Name	Forward Primers (5′-3′)	Reverse Primers (5′-3′)	Product Size (bp)
Ep2	ATGCTCCTGCTGCTTATCGT	AGGGCCTCTTAGGCTACTGC	126
Ep4	TGGCTGTCACTGACCTTCTG	TGCATAGATGGCGAAGAGTG	254
Cox1	GTGGCTATTTCCTGCAGCTC	CAGTGCCTCAACCCCATAGT	209
Cox2	GGGCCCTTCCTCCCGTAGCA	CCATGGCCCAGTCCTCGGGT	232
15-Pgdh	AGGTAGCATTGGTGGATTGG	CCACATCACACTGGACGAAC	105
CD68	TTCTGCTGTGGAAATGCAAG	AGAGGGGCTGGTAGGTTGAT	241
Fst	TGACAATGCCACATACGCCA	CCTCCTCCTCCTCTTCCTCC	131
Mstn	TCAGACCCGTCAAGACTCCT	GGTCCTGGGAAGGTTACAGC	253
Il1β	ACTCATTGTGGCTGTGGAGA	TTGTTCATCTCGGAGCCTGT	199
Il-6	CCGGAGAGGAGACTTCACAG	CAGAATTGCCATTGCACAAC	134
Runx2	CCCAGCCACCTTTACCTACA	TATGGAGTGCTGCTGGTCTG	150
Osx	ACTCATCCCTATGGCTCGTG	GGTAGGGAGCTGGGTTAAGG	238
Ctsk	CCAGTGGGAGCTATGGAAGA	TGGTTCATGGCCAGTTCATA	159
Trap	CAGCAGCCAAGGAGGACTAC	ACATAGCCCACACCGTTCTC	190
Gapdh	CCGGGGCTGGCATTGCTCTC	GTGTTGGGGGCCGAGTTGGG	190
Pax7	GACTCCGGATGTGGAGAAAA	GAGCACTCGGCTAATCGAAC	145
Fndc5	CACGCGAGGCTGAAAAGATG	ACACCTGCCCACATGAAGAG	130

## Data Availability

All original data can be made available upon query or request from the corresponding authors.
